# Rhinophyma Treatment with Blue Laser

**DOI:** 10.22038/ijorl.2025.83754.3818

**Published:** 2025

**Authors:** Estefanía Miranda, Ithzel-María Villarreal, Oscar Arenas, Guillermo Plaza

**Affiliations:** 1 *Department of Otorhinolaryngology Head and Neck Surgery, Faculty of Medicine, Hospital Universitario de Fuenlabrada, Universidad Rey Juan Carlos. Madrid, Spain.*; 2 *Department of Otorhinolaryngology Head and Neck Surgery, Faculty of Medicine, Hospital Universitario Nstra Sra del Rosario, Madrid, Spain. *; 3 *Department of Otorhinolaryngology Head and Neck Surgery, Faculty of Medicine, Hospital Universitario Sanitas La Zarzuela. Madrid, Spain.*

**Keywords:** Blue laser, Rhinophyma, Rosacea, laser therapy

## Abstract

**Introduction:**

Rhinophyma is a chronic skin pathology that mainly affects the nose. It is featured by thickening of the skin and soft tissue of the nose. Treatment options include topical medication, systemic drugs, electrocautery, cryosurgery, laser therapy, dermabrasion, and in some cases, surgical procedures like rhinoplasty. The aim of this report is to demonstrate the usefulness of the blue laser and its efficacy in the treatment of rhinophyma.

**Case Report:**

We report two cases of patients diagnosed with rhinophyma who underwent blue laser treatment.

**Conclusions:**

The use of blue laser to treat rhinophyma has shown to be an effective and safe procedure with very promising results.

## Introduction

Rhinophyma is a chronic skin pathology that mainly affects the nose. It presents by a progressive thickening of the skin and soft tissues of the nose resulting in changes that may lead to nasal airway obstruction marked by external nasal valve collapse and consequently a loss of support of the lower two-thirds of the nose. In most cases, bone and cartilage structures are not affected (1,2). 

Treatment of rhinophyma includes topical medication, systemic drugs and may also include different procedures such as electrocautery, cryosurgery, laser therapy, dermabrasion and in more advanced cases, rhinoplasty.

### Technique

The proper selection of patients is paramount. Our aim was to take advantage of the great affinity of the 450 nm wavelength of this type of laser to red structures, hence we selected patients whose condition already involved the presence of telangiectasias. A consent form was signed by each patient prior to the procedure.

Rhinophyma treatment with blue laser can be performed under general anesthesia or local anesthesia (video 1) (video 2), in case of local anesthesia, topical anesthesia was applied at the office before injection (EMLA: lidocaine and prilocaine). Prior to the procedure, aseptic techniques were performed with aqueous chlorhexidine. The patient and all the operating personnel were given protective eyewear specific for the 450 nm wavelength.

**Fig 1:**
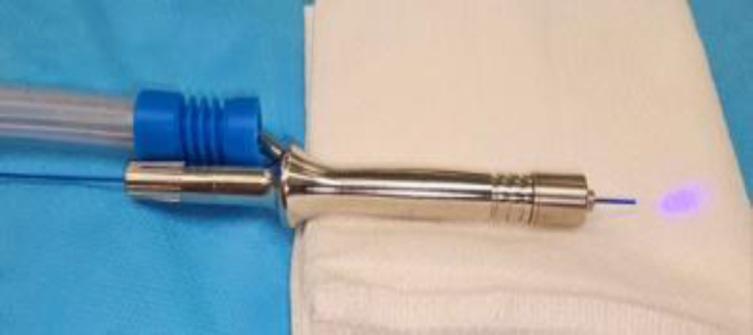
Blue laser surgical handpiece

The blue laser parameters are selected with a manually controlled surgical handpiece (Figure 1) set at 6W power with 50 mS window and 5mS pause. The fiber used measured 600 nm diameter. The number of sessions may vary for each patient. Results should be evaluated by a medical professional at each visit.

**Video 1:**
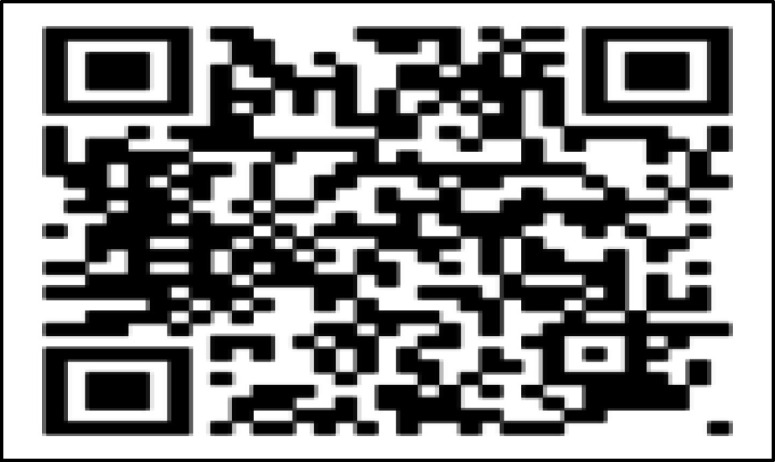
In office procedure with local anesthesia.

**Video 2:**
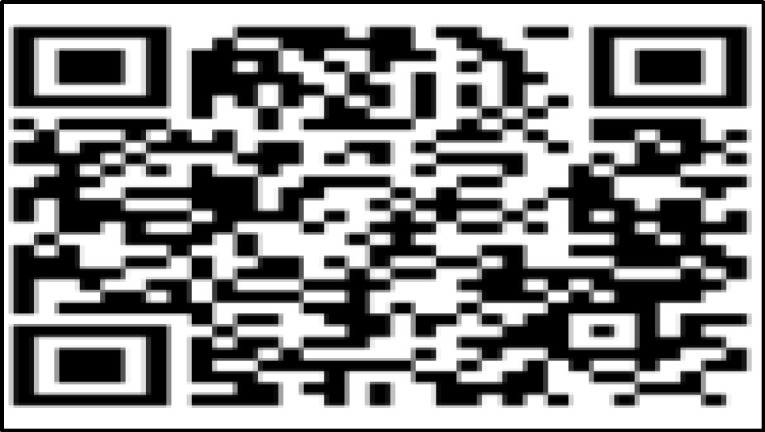
Procedure with general anesthesia.

Post-treatment pain was minimal. Our patients required only Acetaminophen. Re- epithelialization takes 2 to 3 weeks, but erythema may persist for a month. Both of our patients demonstrated significant improvement in skin texture and aesthetic appearance of their noses following the first session.

## Case Report

### Case 1:

A 73-year-old patient presented with bilateral nasal respiratory insufficiency. Physical examination revealed severe left septal deviation, bilateral external valve collapse leading to a severe loss of support in the lower third of the nose and rhinophyma. We decided to perform a functional septorhinoplasty with an external blue laser approach to treat the rhinophyma. 

During septorhinoplasty under general anesthesia the cartilaginous deviation was corrected, ANSA banner and alar rim grafts were placed. Afterwards rhinophyma treatment was performed with blue laser. One month after surgery patient reported significant functional and aesthetic improvement. 

**Fig 2:**
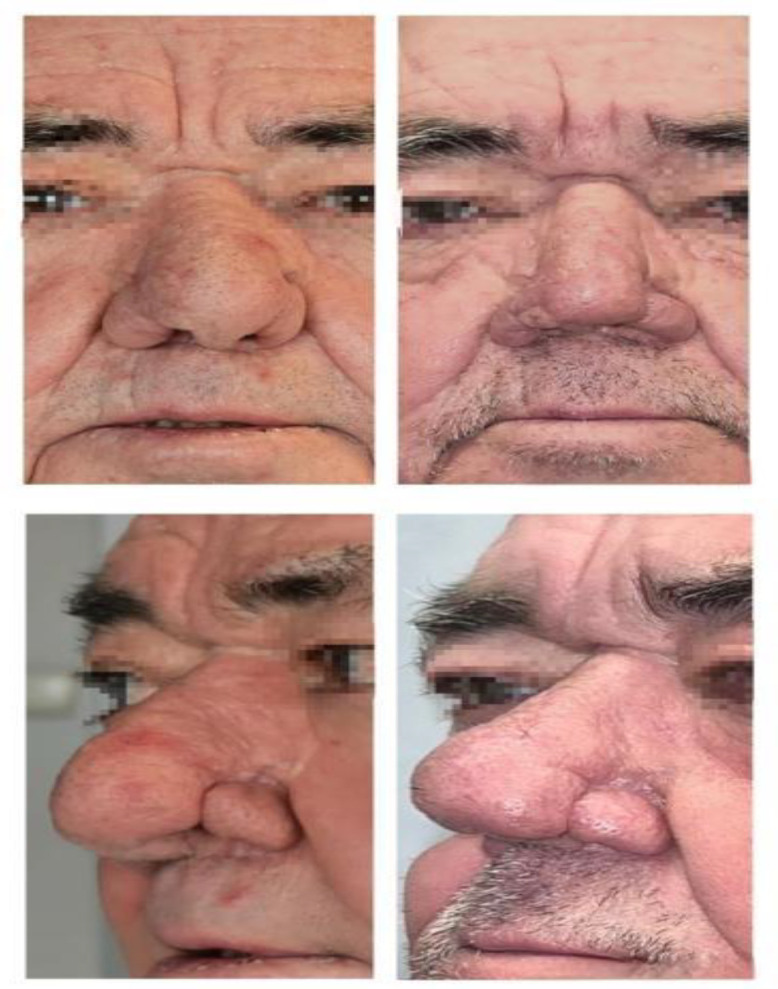
Preoperative and 4-week postoperative images

A new blue laser session was performed in office under local anesthesia without complications and with adequate pain control. Four weeks after the last session, there was evidence of improvement in the telangiectasias, thickness and texture of the skin of the nose (Figure.2).

### Case 2:

A 53-year-old patient with a history of rosacea presents with skin thickening and increase in the size of the nose. Physical examination revealed no septal deviation and no valvular collapse. Nevertheless, telangiectasias, thick skin was observed. Blue laser treatment of his rhinophyma was proposed. 

**Fig 3:**
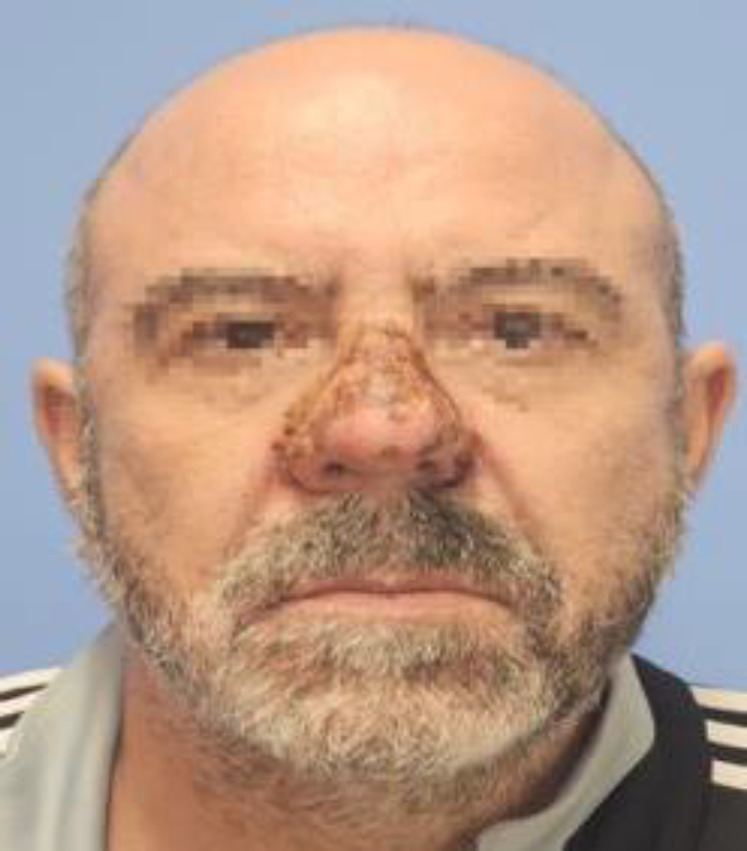
One week after blue laser treatment

Blue laser treatment was performed in office under topical anesthesia. The patient was evaluated one week later showing evidence of scab formed all over the lower two-thirds of the nose (Figure.3). Three weeks later resolution of the healing process was observed and no scabs or erythema were shown. Adequate pain control was assessed. Patient´s satisfaction with the improvement of the external appearance of his skin was documented.

## Discussion

Rhinophyma is regarded as a severe stage of rosacea. It is a disfiguring affection characterized by progressive increased in size and distortion of the external nasal tissue. This condition can lead to various concerns, including functional impairments, aesthetic challenges, and psychosocial issues for affected individuals.

The prevalence of rosacea is 5.46% affecting women more than men. Unlike rosacea, rhinophyma is more frequent in men, and is thought to be caused by increased androgenic activity in males (1,2). 

Rhinophyma affects most frequently Caucasian males in their fifth to seventh decade of life and is uncommon in the African-American and Asian population (1-4). Our patients were both male, one in his fifth and the other one in his seventh decade.

Various treatment modalities have been employed to manage rhinophyma. Among them is laser treatment. Usually, an ablative method which reduces and reshapes the nose is the ideal option. Carbon dioxide (CO_2_), erbium:YAG (Er:YAG), and neodymium YAG (Nd:YAG) laser therapy have been used and more recently blue laser as well obtaining optimal results. 

CO_2_ laser emits invisible light at a wavelength of 10,600 µm, with water as target chromophore. This property allows for removal of tissue via vaporization to be executed^3^. The CO2 laser, with its greater thermal conduction, will result in deep thermal injury to the surrounding tissue with the risk of scarring, rough texture and pigmentation changes and prolonged recovery time but as an advantage it offers excision with minor blood loss and, consequently, provides a predictable esthetic and functional result with precise decortication and controlled hemostasis (1,5-8). 

A laser alternative for ablation is Erbium: YAG (Er: YAG). Er: YAG is a is a solid-state laser that emits infrared light at a wavelength of 2940 nm. It is specific in water absorption and, therefore, gives a better tissue ablation control and minor thermal damage to nearby tissue, leading to a faster recovery time compared to CO2 lasers (5,9). It provides an outstanding option for gentle ablative removal of superficial layers with optimal preservation of surrounding tissue structures. However, the limitations of this approach are bad intraoperative hemostasis, which reduces visualization (10). On the other hand, neodymium: YAG (Nd: YAG laser) laser is a solid-state laser that emits infrared light at a continuous wavelength of 1064 nm, which is absorbed by hemoglobin resulting in the destruction of the vessels. 

Advantages include minimal blood loss, outstanding visualization of tissue planes, and excellent healing (1,11,12).

More recently the development of other lasers has been used for both rosacea and rhinophyma. Vascular lesions have been commonly managed with green (KTP) and yellow (dye) lasers (13). Hemoglobin and melanin, on the other hand, absorb blue light of 420-450 nm better than green or yellow light (14). The advent of blue laser therapy represents a promising advancement. Blue laser emits a wavelength selectively absorbed by blood vessels and sebaceous glands, allowing for precise tissue ablation and remodeling. Blue light can be applied to reduce acne, decrease sebaceous hyperplasia and promote hair growth (15-17). The advantages of blue laser therapy include its minimally invasive nature due to the very low penetration of this wavelength, precise tissue targeting, and favorable cosmetic outcomes. The described therapeutic effect includes the reduction of vascular abnormalities (18). The favorable safety profile of the blue laser is a particular benefit. Another advantage worth mentioning is the portability of the device, with the size of a shoebox, unlike other machines. 

Overall, blue laser therapy represents a promising and effective treatment modality for rhinophyma offering significant improvements in nasal aesthetics and patient´s satisfaction. However, in the current literature there are still no reported cases of treatment with blue laser for this condition. 

Further research is needed to obtain long-term results and comparative effectiveness with other treatment modalities.

Finally, indications for the association of a surgical approach such as rhinoplasty are the improvement of the aesthetic appearance of the deformity and secondary obstruction of the nasal airway, if present. 

In our first case, it was necessary to perform a rhinoplasty due to the loss of support of the lower two thirds of the nose secondary to his rhinophyma.

## Conclusion

Blue laser is an effective, safe and promising technique to treat rhinophyma. Rosacea must be treated in its first stages to minimize any progression to rhinophyma; thus, early recognition and treatment is a critical part of treatment. There are numerous therapeutic modalities for the treatment of rhinophyma. To our knowledge, there are no randomized, prospective, control studies comparing treatments, which makes it challenging to recommend one treatment over another. However, further research is needed to clarify optimal management protocols and long-term outcomes, and comparative effectiveness with other treatment modalities.

The authors declare no potential conflicts of interest, authorship, and/or publication of this article.
